# Association between sustained moderate hyperglycemia within first 48 hours and poor functional outcome after polytrauma: A retrospective cohort study

**DOI:** 10.1007/s00068-026-03099-3

**Published:** 2026-02-23

**Authors:** Matthias Manfred Deininger, Paul Wassersteiner, Nico Haehn, Judith Huth, Gernot Marx, Christian David Weber, Frank Hildebrand, Tim-Philipp Simon, Carina Benstoem, Thomas Breuer

**Affiliations:** 1 Department of Intensive Care Medicine, Faculty of Medicine, RWTH Aachen University, Pauwelsstr. 30, Aachen, 52074 Germany; 2 Department of Orthopaedic, Trauma and Reconstructive Surgery, Faculty of Medicine, RWTH Aachen University, Aachen, Germany

**Keywords:** Polytrauma, Critical care, Stress hyperglycemia, Time-unified hyperglycemic rate, Glasgow outcome scale

## Abstract

**Purpose:**

Stress-induced hyperglycemia is a frequent metabolic response to polytrauma. To date, studies primarily analyzed its association with mortality; however, its effect on functional outcomes remain unclear. This study investigated the association between sustained hyperglycemia across metabolic phases and functional outcomes in polytraumatized ICU patients.

**Methods:**

This retrospective single-center observational study included 176 adult ICU polytrauma patients admitted to a German level 1 university trauma center (2013–2023). Blood ​glucose was quantified using admission values, time-weighted averages, variability and time-unified hyperglycemic rate (TUHyperR; cutoffs 140/160/180 mg/dL) across the early “ebb” (≤ 48 h) and later “flow” (> 48 h) metabolic phases. Primary endpoint was functional outcome using Glasgow outcome scale (GOS; unfavorable GOS ≤ 3). Temporal trends were analyzed using mixed-effects models, and associations with outcomes were assessed using multivariable logistic regression.

**Results:**

Patients with unfavorable outcomes (39.2%) had higher cumulative hyperglycemic exposure throughout their ICU stay. Hyperglycemia was consistently higher during ebb than flow phase. Moderate hyperglycemia during the ebb phase was also in multivariable analysis most discriminative: TUHyperR>140 mg/dL (OR 1.015, 95%-CI [1.004–1.025], *p* = 0.008) and TUHyperR>160 mg/dL (OR 1.016, 95%-CI [1.003–1.030], *p* = 0.017) were independently associated with unfavorable outcomes, whereas TUHyperR>180 mg/dL, admission, mean glucose values as well as glycemic variability were not.

**Conclusion:**

Prolonged moderate hyperglycemia within the first 48 h after polytrauma was independently associated with poor functional outcome at hospital discharge. These exploratory findings support the value of cumulative glycemic exposure as a potential risk marker and highlight the need for prospective studies to determine whether time-based glucose metrics might improve risk stratification and guide metabolic management in polytrauma ICU patients.

**Graphical Abstract:**

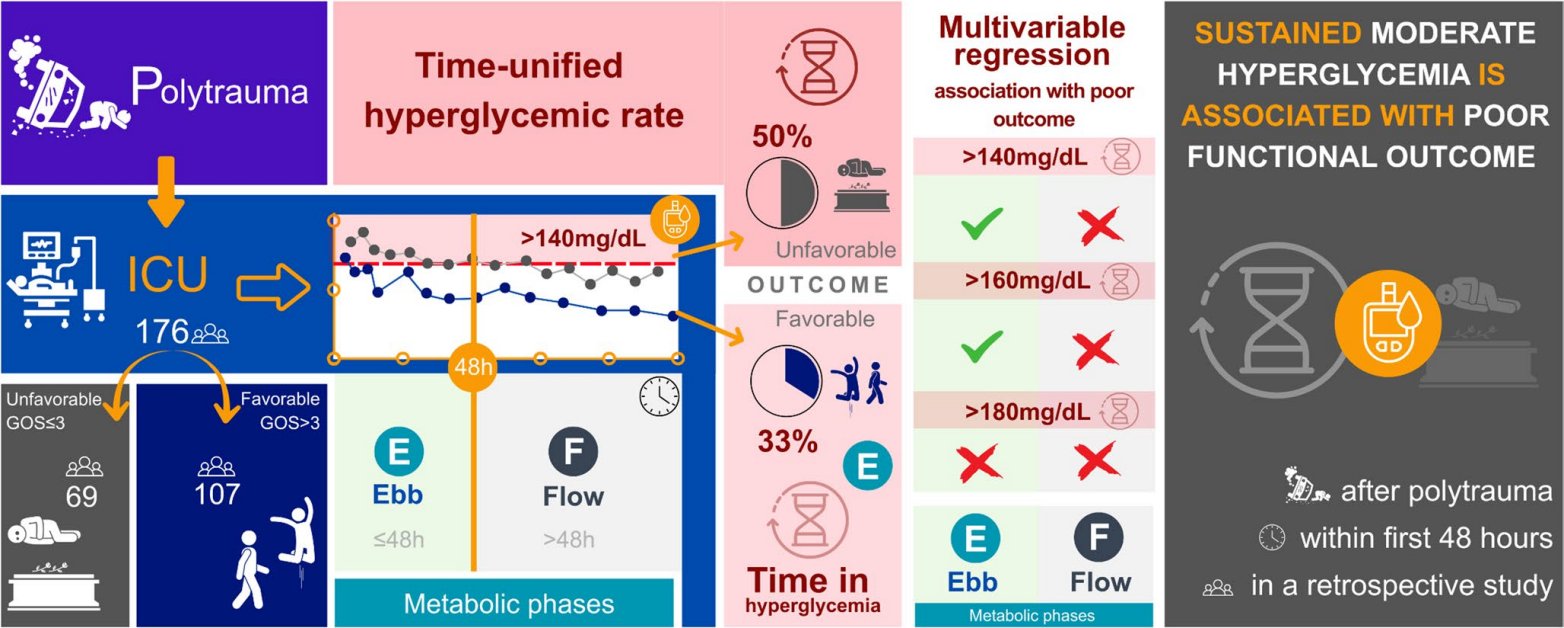

**Supplementary information:**

The online version contains supplementary material available at 10.1007/s00068-026-03099-3.

## Introduction

Trauma is a leading cause of death and disability worldwide [1]. Polytrauma patients with multiple potentially life-threatening injuries are particularly at risk [2, 3]. Despite significant advances in trauma and intensive care management in recent years, trauma-induced metabolic and pathophysiological disturbances challenge outcome prediction [4].

Stress-induced hyperglycemia frequently occurs posttraumatic as a result of elevated levels of glucocorticoids, catecholamines, and proinflammatory cytokines [4, 5]. It is characterized by an increase in blood glucose via induction of gluconeogenesis and insulin resistance. Goal is to ensure substrate availability [6]; however, persistent hyperglycemia results in increased morbidity and mortality [7]. This association is especially pronounced in patients without pre-existing diabetes, highlighting distinct pathophysiological mechanisms of stress-induced versus diabetic hyperglycemia [8, 9]. Severe trauma induces a biphasic systemic stress response, divided into the early “ebb” (≤ 48 h) and the later “flow” (> 48 h) phase [6, 10]. Polytrauma patients suffer from pronounced tissue injury, hypoperfusion, and inflammation. This results in endothelial and immune dysfunction, as well as oxidative stress [4, 11]. Hence, the further amplification of posttraumatic metabolic instability leads to pronounced early stress hyperglycemia during the ebb phase [6]. This early phase is characterized by hypometabolism and reduced oxygen consumption [6, 10]. The subsequent flow phase is a hypermetabolic state with increased oxygen use and metabolic turnover [6, 12]. However, the prognostic relevance of these transient metabolic fluctuations remains unclear.

Most prior studies have focused on admission or mean glucose values as well as glycemic variability in correlation to mortality, neglecting the temporal dynamics of glucose exposure and its potential impact on functional outcome [8, 13–15]. Furthermore, the glycemic cutoffs used to define hyperglycemia vary widely (100–200 mg/dL), mostly all trauma patients were included [16–18] and only few analysis have applied time-resolved indices that capture both the magnitude and duration of hyperglycemia [19]. Established indices for quantifying hyperglycemia over time in ICU, such as time-over-range or hyperglycemic index [20], are directly influenced by heterogeneous measurement frequency and have rarely been evaluated in trauma populations [19]. In the present study, the time-unified hyperglycemic rate (TUHyperR) was used as it quantifies the proportion of predefined time periods spent in hyperglycemia and is thus less vulnerable to irregular or varying sampling frequencies [21].

To date, little is known about the correlation between sustained hyperglycemia and functional outcomes after polytrauma. To address this gap, we conducted an exploratory, retrospective, single-center study of ICU-treated, adult polytrauma patients to evaluate the value of sustained hyperglycemic burden on functional outcomes, separated by metabolic stress response phases.

## Methods

### Study design

This retrospective, single-center observational study was conducted in accordance with the Strengthening the Reporting of Observational Studies in Epidemiology (STROBE) guidelines [22]. The study was approved by the Ethics Committee of the Medical Faculty of RWTH Aachen University, Germany (approval number: EK23-216). Due to the retrospective and anonymized data analysis, written informed patient consent was waived. All research procedures complied with the ethical standards of the institutional review board and the applicable guidelines and regulations, including the Declaration of Helsinki. The primary endpoint of this study was unfavorable outcome on hospital discharge, defined as Glasgow outcome scale (GOS) ≤ 3 (severe disability, vegetative state, or death) [23]. GOS represents a validated five-point functional outcome measure ranging from 1 (death) to 5 (good outcome).

### Patient selection

All trauma patients admitted to the trauma bay of the level-1 trauma center RWTH Aachen University Hospital, Germany between 2013 and 2023 were screened, irrespective of injury mechanism (blunt and penetrating trauma). Inclusion further required admission to the ICU, a minimum ICU length of ≥ 72 h to ensure adequate temporal resolution for glycemic analysis and fulfillment of the latest, so-called ‘Berlin definition’ of polytrauma. The latter defines polytrauma as injuries in at least two different body regions (Abbreviated injury scale, AIS ≥ 3), accompanied by at least one of the following: hypotension (systolic blood pressure < 90 mmHg), unconsciousness (GCS ≤ 8), acidosis (base excess ≤ − 6.0 mmol/L), coagulopathy (aPTT > 40 s or INR > 1.4) or age ≥ 70 years [2]. Exclusion criteria comprised: pediatric patients (< 18 years), burn patients, patients admitted later than 6 h post-injury, patients with pre-existing diabetes mellitus and missing GOS on hospital discharge.

### Data collection

Demographics, medical history, and clinical patient data were gathered from institutional electronic medical records and the ICU patient data management system (IntelliSpace Critical Care and Anesthesia, Koninklijke Philips N.V., Amsterdam, Netherlands, version J.05.01; Medico, CompuGroup Medical SE & Co. KGaA, Koblenz, Germany, version 29.00.02.00).

Data collection included (a) demographic data: age, sex, body mass index (BMI); (b) medical history: diabetes mellitus, hypertension, coronary heart disease, peripheral arterial disease, asthma bronchiale, chronic obstructive pulmonary disease (COPD), chronic kidney disease, alcohol abuse, drug abuse, smoking history; (c) scores: injury severity score (ISS), simplified acute physiology score II (SAPS II); (d) injured regions according to AIS: head and neck, face, chest, abdomen, extremities (including pelvis), soft tissue and respective AIS severity level; (e) ICU treatment and result: ventilator support, duration of mechanical ventilation, frequency of blood gas analysis per 24 h, catecholamine use, transfusion, ICU and hospital mortality, ICU and hospital length of stay (LOS); (f) complications during ICU: wound infection, urinary tract infection, pneumonia, acute respiratory distress syndrome (ARDS), sepsis, acute kidney failure, dialysis, delirium; (g) primary outcome: GOS at hospital discharge.

### Data extraction of blood glucose values

Only blood gas measurements using point-of-care blood gas analyzers (ABL90 FLEX, Radiometer, Krefeld, Germany) were included, as these provide exact timestamps. Data were retrieved from the institutional data warehouse (SAP SE, Walldorf, Germany, version 14.2.6.2953) and processed using RStudio (Posit PBC, Boston, MA, USA; version 2025.05.0 + 496) with R (version 4.5.1).

### Definitions of glycemic indices

**Fig. 1:**
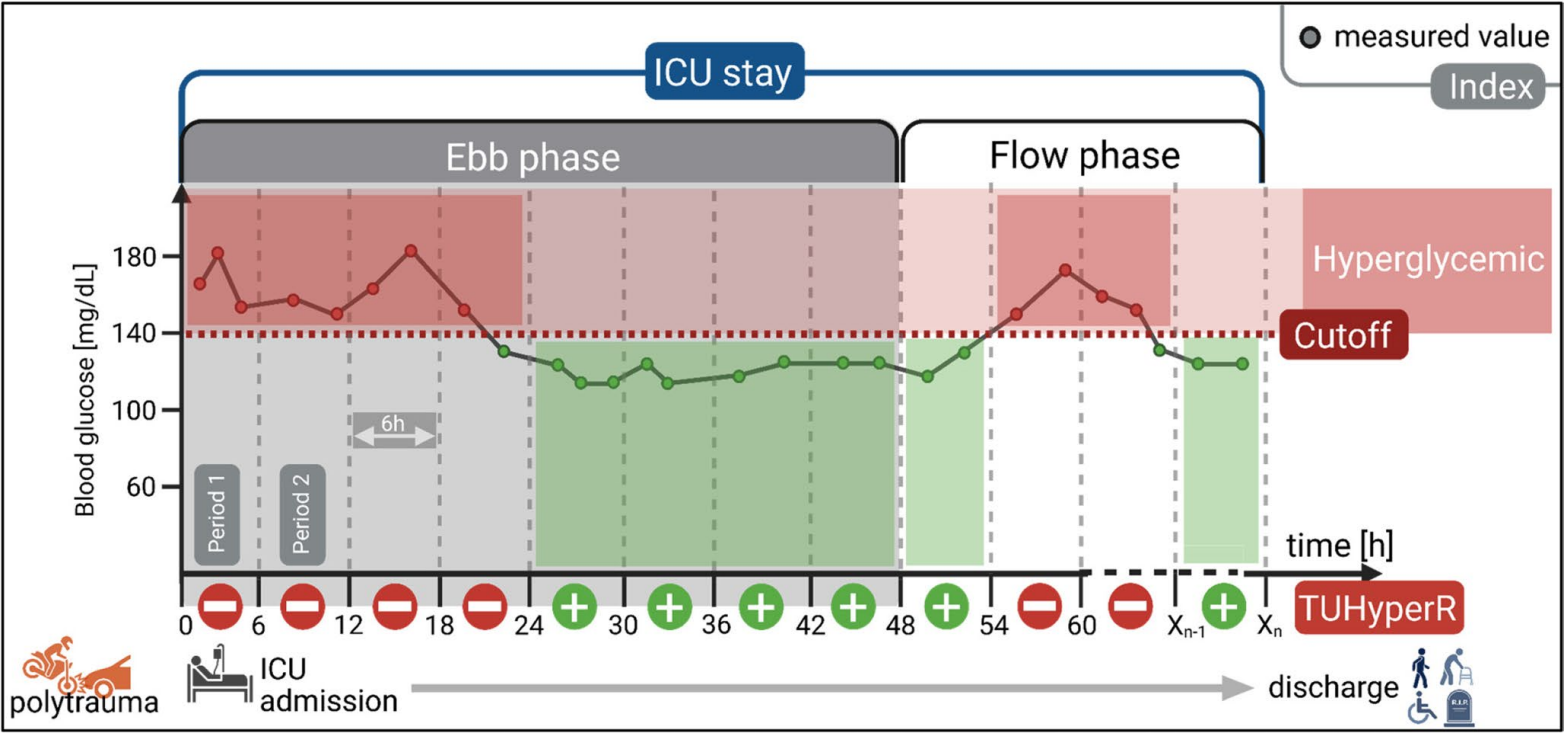
Calculation of time-unified hyperglycemic rate (TUHyperR). Calculation principle of TUHyperR is illustrated in this figure for a fictional example patient. Abscissa represents ICU timeline, ordinate blood glucose levels. ICU stay was divided into time periods (here: 6 h each) for subsequent TUHyperR calculation. The ratio between the number of periods containing hyperglycemic values (cutoff here 140mg/dL) to the total number of periods is calculated, to obtain TUHyperR. In this example, four of the eight periods in the ebb phase (≤48h) included hyperglycemic values, resulting in a TUHyperR of 50%. TUHyperR was calculated separately for each patient over the entire stay, as well as for ebb and flow phases. Created with BioRender. Deininger, M. (2025) https://BioRender.com/l7qhnh5; ICU: Intensive care unit

Owing to the retrospective nature of the study design, the frequency and timing of blood glucose measurements varied among patients. Relying exclusively on single or average values might introduce bias towards periods with frequent short-term sampling, potentially obscuring significant trends. Hence, to robustly describe glycemic status over time, the following indices were calculated for each patient:

- Admission blood glucose value (ABG) is the first blood glucose measurement after ICU admission.
- Mean blood glucose (MBG) and time-weighted average (TWAG). MBG indicates the arithmetic absolute mean of all measured values. TWAG incorporates the temporal distribution of values and is calculated using the trapezoidal rule, in which the area under the measurement curve is divided by the total observation time [20].
- Glycemic variability (CVG) is described by the coefficient of glycemic variation calculated by dividing the standard deviation by the MBG [24].
- Time-unified hyperglycemic rate (TUHyperR) was used to quantify time duration above the hyperglycemic cutoff (140, 160, 180 mg/dL, Fig. 1). The calculations are described in detail elsewhere [21]. In brief, the ICU stay is divided into equal-length periods, starting with admission (period length assessed: 1, 2, 3, 4, 6, 8, and 12 h). The optimal period length is determined by balancing the highest possible measurement quality with temporal resolution. Therefore, the shortest period length is chosen, containing at least one measured value per patient in median > 95% of all periods. This period length is used for all patients. TUHyperR was calculated for each patient individually by dividing the number of hyperglycemic periods by the total number of periods containing at least one measurement value [21].

All indices were calculated, in addition to the entire ICU stay, separately for the early ebb and later flow metabolic phases. Hyperglycemic cutoffs (> 140, 160, 180 mg/dL) for TUHyperR were prespecified to span moderate-to-pronounced hyperglycemia within commonly used ranges. The 180 mg/dL cutoff reflects a widely recommended trigger for initiating management of persistent hyperglycemia in critically ill adults, whereas 140 and 160 mg/dL were selected to represent moderate hyperglycemia below this treatment cutoff frequently used in time-based glucometrics and time-in-range concepts [25–27]. These cutoffs were used to characterize glycemic exposure and were not intended to define therapeutic targets or a single optimal prognostic cutoff.

### Standard treatment of polytrauma patients

All patients received standardized trauma and intensive care in accordance with national (German S3-guidelines) and international recommendations [28–30]. Initial treatment in the preclinic and emergency department followed established prehospital and advanced trauma life support principles, including airway management, hemorrhage control, early imaging, and goal-directed resuscitation [31]. Upon ICU admission, treatment focused on hemodynamic stabilization, organ support, and prevention of secondary injuries [32]. ​Blood glucose levels were intermittently monitored, and intravenous insulin infusion was initiated for persistent hyperglycemia exceeding 180 mg/dL, targeting a range of 140–180 mg/dL, in accordance with the Society of Critical Care Medicine (SCCM) guidelines [25, 33]. Nutritional support followed European Society for Clinical Nutrition and Metabolism and German Society for Nutritional Medicine recommendations, with early enteral feeding initiated within 24–48 h as clinically feasible [33, 34].

### Statistics

All statistical analysis were performed using IBM SPSS Statistics (IBM Corporation, Armonk, NY, USA, version 29.0.2.0(20)) and RStudio (Posit PBC, Boston, MA, USA; version 2025.05.0 + 496) with R version 4.5.1.

Normality was assessed using the Shapiro-Wilk test. As all values were non-normally distributed, continuous variables are reported as medians and interquartile ranges (IQR). Categorical variables are presented as absolute numbers and percentages. Group comparisons were conducted using the Mann-Whitney U test for continuous and Chi square test or Fisher’s exact test for categorical variables. Paired data were analyzed using Wilcoxon signed-rank test. All tests were two-tailed, and a p-value < 0.050 was considered statistically significant.

To analyze glucose dynamics over time, linear mixed-effects models with restricted cubic splines (four degrees of freedom) were applied. Glycemic values were calculated using TWAG, as time period length, the one identified as optimal for TUHyperR, was applied. Model complexity was assessed by comparing the Akaike information criterion values across spline models with two to seven degrees of freedom, with the four degree model offering an optimal compromise between fit quality and model simplicity. Models included fixed effects for time and GOS-groups (favorable, GOS > 3 vs. unfavorable, GOS ≤ 3), as well as random intercepts and slopes for each spline component at patient level to account for inter-individual variability. Nonlinear time trends and group differences were tested using likelihood ratio test.

Associations between glycemic indices and unfavorable functional outcome were assessed using univariable and multivariable logistic regression analysis. Variables with *p* < 0.050 in the univariable analysis were included in multivariable models adjusted for age, sex, and ISS. Sensitivity analyses were conducted to test robustness by adjusting for head injury severity given its expected major impact on functional outcomes, either as any head injury (AIS head > 0), severe head injury (AIS ≥ 3), or as a continuous AIS head score. Further sensitivity analyses were performed for ISS body regions showing significant between-group differences in AIS severity to address potential confounding. Odds ratios (OR) with 95% confidence intervals (CI) were reported. Figure 1 was created using BioRender.com. Figures 3 and 4 and S1 were created using Prism (GraphPad Software Inc., San Diego, USA, version 10.6.1).

## Results

### Study population and patient characteristics

**Fig. 2:**
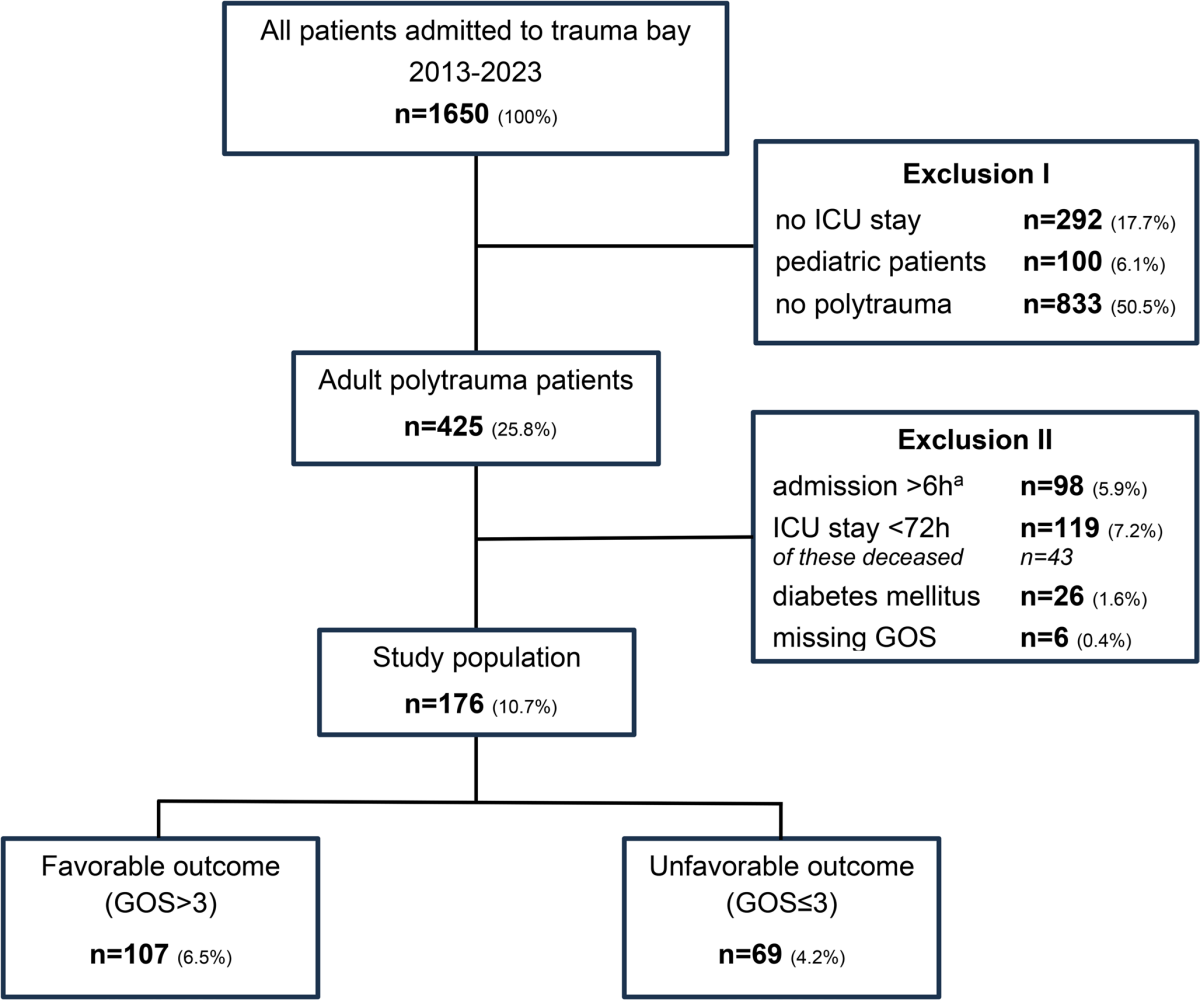
Study population flow chart. Absolute number and percentage share relative to all screened patients are shown. GOS: Glasgow outcome scale, ICU: Intensive care unit. ^a^ secondary transferred patients from other hospitals for further treatment at our university hospital

A total of 176 adult polytrauma patients met the inclusion criteria (Fig. 2). The median age was 49 years (28–64), and 75.0% were male. Based on the GOS at hospital discharge, 107 patients (60.8%) achieved a favorable outcome (fav, GOS >3), the resulting 69 patients (39.2%) an unfavorable outcome (unfav, GOS ≤3).

Baseline demographics, medical history, SAPS II score and length of ICU as well as hospital stay were broadly comparable between both GOS groups (Table 1). With regard to incidence and median AIS severity of injuries by ISS region, no between-group differences were found, except for chest injury severity (fav: 3.0 (2.0–3.0), unfav: 3.0 (3.0–4.0), *p* = 0.020; Supplement, Table S1). Moreover, no difference was observed in the occurrence of ventilation, transfusion, and catecholamine therapy during the ICU stay.

**Table 1:** Patient characteristics

Variable	Study population	Hospital discharge Glasgow outcome scale		
		Favorable (>3)	Unfavorable (≤3)	*p*-value
	*n* = 176	*n* = 107 (60.8%)	*n* = 69 (39.2%)	
DEMOGRAPHY				
Age [years]	49.0 (28.0–64.0)	49.0 (27.0–64.0)	48.0 (30.0–65.0)	0.580
Sex [male]	132 (75.0%)	79 (73.8%)	53 (76.8%)	0.723
BMI [kg/m²]	25.7 (23.9–27.8)	26.0 (24.2–29.2)	24.9 (23.1–27.7)	0.148
MEDICAL HISTORY [*n*, %]				
Hypertension	26 (14.8%)	16 (15.0%)	10 (14.5%)	1.000
Coronary heart disease	4 (2.3%)	3 (2.8%)	1 (1.4%)	1.000
Peripheral artery disease	3 (1.7%)	3 (2.8%)	0 (0.0%)	0.281
Asthma	5 (2.8%)	3 (2.8%)	2 (2.9%)	1.000
COPD	4 (2.3%)	2 (1.9%)	2 (2.9%)	0.645
Chronic kidney disease	3 (1.7%)	2 (1.9%)	1 (1.4%)	1.000
Alcohol abuse	17 (9.7%)	12 (11.2%)	5 (7.2%)	0.444
Drug abuse	12 (6.8%)	9 (8.4%)	3 (4.3%)	0.370
Smoking history	14 (8.0%)	8 (7.5%)	6 (8.7%)	0.782
TRAUMA KEY FACTS				
ISS	34.0 (27.0–38.0)	29.0 (26.0–36.0)	34.0 (29.0–43.0)	**< 0.001**
ISS regions affected [n, %]^a^				
*Head and neck	137 (77.8%)	80 (74.8%)	57 (82.6%)	0.267
*Face	86 (48.9%)	54 (50.5%)	32 (46.4%)	0.645
*Chest	151 (85.8%)	90 (84.1%)	61 (88.4%)	0.510
*Abdomen	83 (47.2%)	49 (45.8%)	34 (49.3%)	0.757
*Extremities (including pelvis)	142 (80.1%)	86 (80.4%)	56 (81.2%)	0.530
*Soft tissue	36 (20.5%)	19 (17.8%)	17 (24.6%)	0.180
ICU TREATMENT AND OUTCOMES				
SAPS II at ICU admission	31.0 (27.0–37.0)	31.0 (26.0–36.0)	33.0 (28.0–40.0)	0.192
Catecholamine therapy	155 (88.1%)	91 (85.0%)	64 (92.8%)	0.156
Transfusion in ICU	77 (43.8%)	41 (38.3%)	36 (52.2%)	0.087
BGA frequency per 24 h	7.4 (6.2–8.7)	6.9 (6.0–8.0)	8.5 (7.0–9.6.0.6)	**< 0.001**
Ventilation	168 (95.5%)	101 (94.4%)	67 (97.1%)	0.484
Ventilation duration [hours]	293.0 (61.0–645.0)	166.0 (44.0–503.0)	408.0 (182.0–846.0)	**< 0.001**
ICU LOS [days]	19.0 (9.1–34.1)	17.8 (8.8–30.9)	20.8 (11.9–40.2)	0.060
Hospital LOS [days]	27.8 (17.0–42.2)	28.5 (20.8–42.8)	25.3 (15.7–41.6)	0.244
ICU mortality	17 (9.7%)	0 (0.0%)	17 (24.6%)	**< 0.001**
Hospital mortality	18 (10.2%)	0 (0.0%)	18 (26.1%)	**< 0.001**
ICU COMPLICATIONS [*n*, %]				
Wound infection	25 (14.2%)	13 (12.1%)	12 (17.4%)	0.379
Urinary tract infection	18 (10.2%)	11 (10.3%)	7 (10.1%)	1.000
Pneumonia	79 (44.9%)	36 (33.6%)	43 (62.3%)	**< 0.001**
ARDS	15 (8.5%)	7 (6.5%)	8 (11.6%)	0.276
Sepsis	43 (24.4%)	21 (19.6%)	22 (31.9%)	0.074
Acute kidney failure	26 (14.8%)	7 (6.5%)	19 (27.5%)	**< 0.001**
Dialysis^b^	12 (6.8%)	3 (2.8%)	9 (13.0%)	**0.011**
Delirium	39 (22.2%)	28 (26.2%)	11 (15.9%)	0.138

Continuous variables are reported as median (IQR) and categorical variables as absolute number (percentage). Significant p-values are shown in bold

*ARDS* Acute respiratory distress syndrome, *BGA* Blood gas analysis, *BMI* Body mass index, *GOS* Glasgow outcome scale, *ICU* Intensive care unit, *ISS* Injury severity score, *IQR* Interquartile range, *LOS* Length of stay, *SAPS II* Simplified acute physiology score II

^a^ detailed distribution of abbreviated injury scale levels for ISS regions in Supplement, Fig. S1

^b^ missing values: dialysis (n=2 in unfavorable group)

However, patients with unfavorable outcomes had significantly higher ISS (fav: 29.0 (26.0–36.0), unfav: 34.0 (29.0–43.0), *p* < 0.001), longer duration of mechanical ventilation (fav: 166.0 (44.0–503.0), unfav: 408.0 (182.0–846.0), *p* < 0.001), higher blood gas measurement frequency per 24 h (fav: 6.9 (6.0–8.0), unfav: 8.5 (7.0–9.6), *p* < 0.001) and higher rates of ICU complications such as acute kidney failure (fav: 6,5%, unfav: 27.5%, *p* < 0.001) or pneumonia (fav: 33.6%, unfav: 62.3%, *p* < 0.001).

### Glycemic indices higher in unfavorable outcome group

Patients with unfavorable outcome had significantly higher mean blood glucose (MBG: fav: 124.3 (117.1–134.2) mg/dL, unfav: 128.7 (119.4–138.9) mg/dL, *p* = 0.039), higher time-weighted average glucose (TWAG fav: 122.5 (115.1–130.5) mg/dL, unfav: 126.4 (118.0–136.2) mg/dL, *p* = 0.019), and longer exposure to hyperglycemia (Table 2).

**Table 2:** Glycemic indices separated by outcome groups

Variable	Study population	Hospital discharge Glasgow outcome scale		
		Favorable (>3)	Unfavorable (≤3)	_*p*-value_
	*n* = 176	*n* = 107	*n* = 69	
ABG [mg/dL]	141.0 (119.0–170.0)	137.0 (119.0–159.0)	145.0 (122.0–177.0)	0.220
MBG [mg/dL]	125.5 (118.1–135.2)	124.3 (117.1–134.2)	128.7 (119.4–138.9)	**0.039**
TWAG [mg/dL]	123.7 (116.1–132.9)	122.5 (115.1–130.5)	126.4 (118.0–136.2)	**0.019**
CVG [%]	15.9 (13.5–19.4)	15.7 (13.3–18.8)	16.7 (13.8–20.3)	0.136
TUHyperR (> 140 mg/dL) [%]	26.5 (13.0–41.2)	22.6 (11.5–37.8)	30.9 (17.2–48.0)	**0.009**
TUHyperR (> 160 mg/dL) [%]	7.1 (1.9–15.2)	6.4 (1.8–13.8)	10.6 (2.4–23.1)	**0.018**
TUHyperR (> 180 mg/dL) [%]	2.0 (0.0–5.9)	1.2 (0.0–4.0)	3.3 (0.6–10.0)	**0.004**

Median (IQR) values are shown separately for favorable (GOS >3) and unfavorable (GOS ≤3) outcome groups. Significant p-values are shown in bold

*CVG* Coefficient of variation for glucose, *GOS* Glasgow Outcome Scale, *IQR* Interquartile range, *MBG* Mean blood glucose, *TUHyperR* Time-unified hyperglycemic rate, *TWAG* Time-weighted average glucose

At first, 6 h was identified as the optimal period length for TUHyperR as described in the methods section (median measurement coverage 1 h: 29.5%; 2 h: 55.5%; 3 h: 75.0%; 4 h: 86.1%; 6 h: 95.8%; 8 h: 98.4%; 12 h: 100.0%). The resulting time in hyperglycemia (TUHyperR>140 mg/dL) was - with about one third of the ICU stay (30.9% (17.2%−48.0%)) - significantly longer for patients with unfavorable outcome compared to approximately one quarter for those with favorable outcome (22.6% (11.5%−37.8%), *p* = 0.009). For higher hyperglycemic cutoffs statistically comparable but absolute lower percentages were seen (TUHyperR cutoff > 160 mg/dL fav: 6.4% (1.8%−13.8%), unfav: 10.6% (2.4%−23.1%), *p* = 0.018; >180 mg/dL fav: 1.2% (0.0%−4.0%), unfav: 3.3% (0.6%−10.0%), *p* = 0.004). Admission blood glucose and glycemic variability did not differ according to the GOS outcome. Given that MBG and TWAG showed absolute as well as statistically comparable results, for improved readability, only the latter is presented for the following analysis. All MBG results are shown in Supplementary, Tables S2-S4, S6.

### Glucose persistently higher during ICU stay for unfavorable outcome

To analyze changes over time subsequently to the overall group differences shown, blood glucose trends were investigated based on TWAG values calculated for 6 h time periods for each patient.

**Fig. 3:**
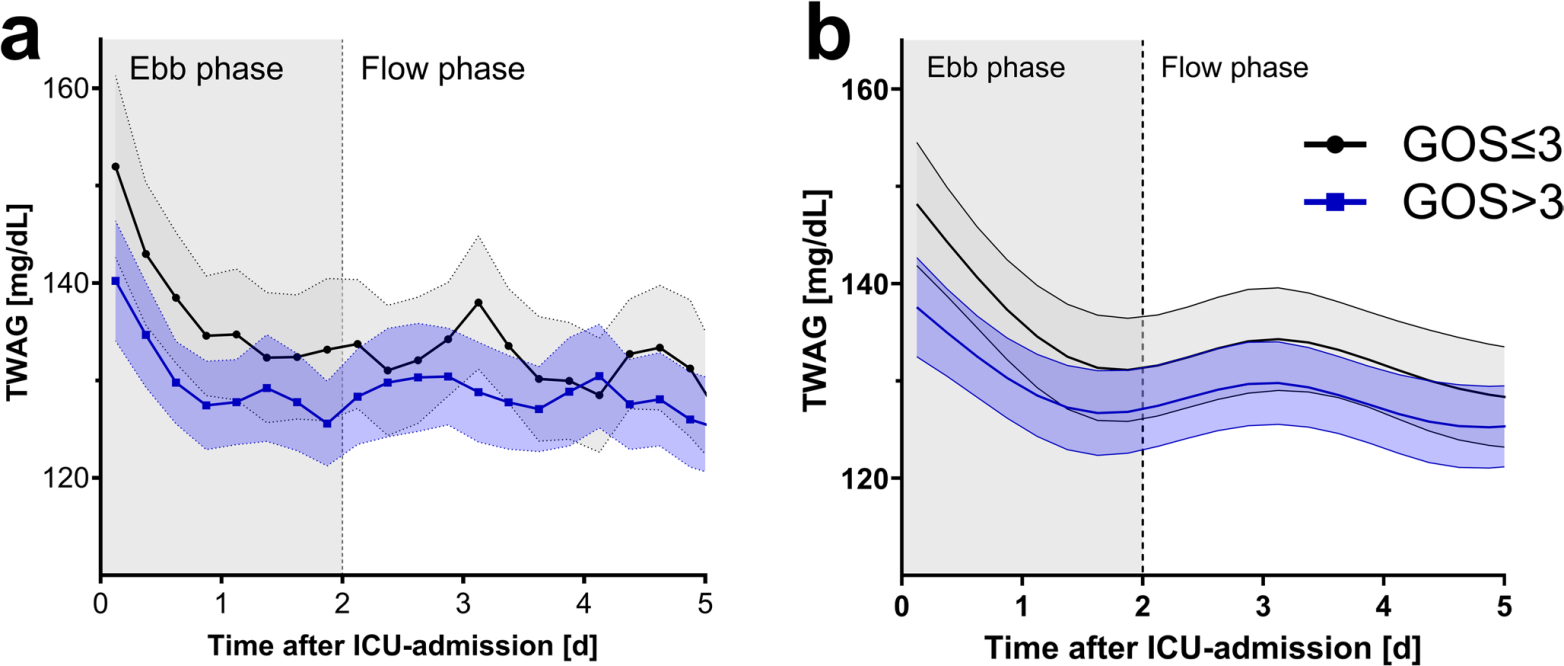
Time course of time-weighted average glucose stratified by outcome. The time course of time-weighted average glucose (TWAG) is depicted over the first five ICU days, stratified by favorable (blue curve, GOS >3) or unfavorable (gray curve, GOS ≤3) outcome. Abscissa represents ICU timeline in days, ordinate the TWAG values calculated for 6 h time periods for each patient. (**a**) shows raw values, (**b**) estimated marginal mean intervals derived from restricted cubic spline mixed model with four degrees of freedom. Shaded ribbons represent 95%-CI. GOS: Glasgow outcome scale

The blood glucose trend showed highest values in both GOS-groups immediately after ICU admission, with a significant drop within the first 48 h (ebb phase) and more stable values thereafter (flow phase, Fig. 3a). Considering the spline-based mixed-effects model, the unfavorable polytrauma group exhibited consistently higher glucose values (*p* = 0.011, Fig. 3b).

### Hyperglycemia most pronounced during ebb phase

For a quantitative analysis of the graphical qualitatively visualized difference over time, blood glucose indices for ebb and flow phases were calculated separately for each GOS-group. In both GOS-groups, glucose values were higher during the ebb phase, with absolutely lower values in the favorable group (Table 3).

**Table 3:** Glycemic indices during ebb and flow phases stratified by outcome group

Variable	Phases		*p*-value
	Ebb	Flow	
Favorable GOS (> 3)			
TWAG [mg/dL]	127.7 (116.8–141.3)	119.3 (111.4–128.1)	**< 0.001**
CVG [%]	14.0 (10.0–18.0)	15.0 (12.0–18.0)	0.085
TUHyperR (> 140 mg/dL) [%]	33.3 (12.5–62.5)	20.0 (9.5–34.7)	**< 0.001**
TUHyperR (> 160 mg/dL) [%]	12.5 (0.0–28.6)	4.9 (0.0–10.8)	**< 0.001**
TUHyperR (> 180 mg/dL) [%]	0.0 (0.0–12.5)	0.0 (0.0–3.6)	**0.003**
Unfavorable GOS (≤ 3)			
TWAG [mg/dL]	137.2 (123.2–150.7)	124.8 (117.1–135.0)	**< 0.001**
CVG [%]	14.0 (11.0–19.0)	16.0 (12.0–20.0)	0.480
TUHyperR (> 140 mg/dL) [%]	50.0 (25.0–87.5)	29.7 (14.3–45.7)	**< 0.001**
TUHyperR (> 160 mg/dL) [%]	25.0 (0.0–50.0)	8.0 (1.1–19.0)	**< 0.001**
TUHyperR (> 180 mg/dL) [%]	12.5 (0.0–14.3)	1.8 (0.0–6.3)	**< 0.001**

Data are analyzed separately for favorable and unfavorable outcome groups. For both groups, glycemic values for ebb and flow phases are examined. Data are shown as median (IQR). Significant p-values are shown in bold

*CVG* Coefficient of variation for glucose, *GOS* Glasgow outcome scale, *IQR* Interquartile range, *TUHyperR* Time-unified hyperglycemic rate, *TWAG* Time-weighted average glucose

**Fig. 4:**
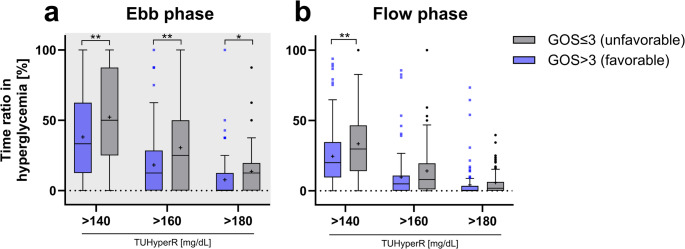
Time-unified hyperglycemic rate for ebb and flow phases separated by functional outcome groups. Time-unified hyperglycemic rate (TUHyperR) is shown for the three different cutoffs (>140, >160 and >180mg/dL) separately for (**a**) ebb and (**b**) flow phase. Data are separated by Glasgow outcome scale (GOS) groups and compared statistically using Mann-Whitney U test. Data are presented as median with interquartile range as box and Tukey whiskers. Data outside the Tukey range are shown as separate dots; mean is shown as +; *p<0.050, **p<0.010

In patients with unfavorable outcome, median TWAG decreased significantly by about 12 mg/dL from the ebb to flow phase (ebb: 137.2 mg/dL, flow: 124.8 mg/dL, *p* < 0.001). Consistently, the duration in hyperglycemia (TUHyperR>140 mg/dL) was significantly reduced from half of the time (50.0% (25.0%−87.5%)) in the ebb phase to about one-third (29.7% (14.3%−45.7%)) in the flow phase (*p* < 0.001) when using 140 mg/dL as cutoff. Comparable differences were observed for cutoffs of 160 mg/dL (ebb: 25.0% (0.0%−50.0%), flow: 8.0% (1.1%−19.0%), *p* < 0.001) and 180 mg/dL (ebb: 12.5% (0.0%−14.3%), flow: 1.8% (0.0%−6.3%), *p* < 0.001). Glycemic variation (CVG) did not differ significantly between the ebb and flow phases or between the two GOS-groups. In the favorable outcome group, glycemic values showed equivalent trends. When comparing the outcome groups for the ebb and flow phases separately, similar results were seen, except for TUHyperR in the flow phase, were only TUHyperR>140 mg/dL showed significant group difference (*p* = 0.009, Fig. 4; Supplementary, Table S4).

### Early sustained hyperglycemia is associated with poor functional outcome

To assess the predictive potential of blood glucose indices, their association with GOS-group was examined. In the univariable regression analysis, significant results were obtained for all glucose indices in the ebb phase, for TUHyperR>160 mg/dL and > 140 mg/dL over the entire period, and for TUHyperR>140 mg/dL also for the flow phase (Table 4).

**Table 4:** Uni- and multivariable logistic regression for outcome association

Variable	Univariable regression		Multivariable regression	
	OR [95%-CI]	*p*-value	OR [95%-CI]	*p*-value
Age^#^	1.004 [0.989–1.019]	0.630	1.002 [0.985–1.019]	0.806
Sex^#^	0.852 [0.420–1.725]	0.656	0.825 [0.387–1.756]	0.617
ISS^#^	1.072 [1.033–1.113]	**< 0.001**	1.074 [1.035–1.116]	**< 0.001**
TWAG				
Full ICU stay	1.018 [0.999–1.037]	0.070		
Ebb phase	1.015 [1.000–1.030]	**0.046**	1.015 [0.999–1.032]	0.070
Flow phase	1.018 [0.999–1.038]	0.064		
TUHyperR (> 140 mg/dL)				
Full ICU stay	1.017 [1.003–1.030]	**0.015**	1.016 [1.000–1.032]	0.050
Ebb phase	1.014 [1.004–1.024]	**0.005**	1.015 [1.004–1.025]	**0.008**
Flow phase	1.016 [1.003–1.030]	**0.016**	1.014 [0.999–1.030]	0.065
TUHyperR (> 160 mg/dL)				
Full ICU stay	1.019 [1.001–1.038]	**0.043**	1.015 [0.995–1.035]	0.149
Ebb phase	1.018 [1.006–1.031]	**0.003**	1.016 [1.003–1.030]	**0.017**
Flow phase	1.015 [0.997–1.034]	0.103		
TUHyperR (> 180 mg/dL)				
Full ICU stay	1.021 [0.993–1.051]	0.144		
Ebb phase	1.022 [1.002–1.042]	**0.029**	1.015 [0.995–1.035]	0.141
Flow phase	1.014 [0.985–1.044]	0.340		

Glycemic indices that showed significant effects in the univariable analysis were included in separate multivariable models. All multivariable logistic regression models were adjusted for the baseline covariates age, sex and ISS. Odds ratios (OR) and 95% confidence intervals (CI) are displayed. Significant p-values are shown in bold

*ISS* Injury severity score, *TUHyperR* Time-unified hyperglycemic rate, *TWAG* Time-weighted average glucose

^#^ For these parameters, OR [95%-CI] is shown exemplarily for multivariable regression with TWAG - ebb phase, although the OR [95%-CI] vary slightly depending on the blood glucose index chosen, the significance in all multivariable regressions was unchanged. The exact data for each of the multivariable regressions are summarized in Supplementary, Tables S5-S7

In multivariable models adjusted for age, sex and ISS, TUHyperR>140 mg/dL during the ebb phase remained independently associated with unfavorable GOS (OR 1.015, 95%-CI [1.004–1.025], *p* = 0.008, Table 4). An increase of TUHyperR>140 mg/dL of 10%, therefore, corresponded to an approximately 15–16% relative increase in the probability of an unfavorable outcome. This association also remained significant for TUHyperR > 160 mg/dL (OR 1.016, 95%-CI [1.003–1.030], *p* = 0.017), but not for TUHyperR > 180 mg/dL (OR 1.015, 95%-CI [0.995–1.035], *p* = 0.141).

In sensitivity analyses additionally adjusting for head and, separately, for chest injury severity, results for TUHyperR>140 mg/dL and > 160 mg/dL remained significant (Supplementary, Tables S8-S10). However, ISS showed the greatest positive association in all multivariable analysis.

## Discussion

In this single-center cohort of critically ill polytrauma patients, sustained moderate hyperglycemia during the first 48 h, corresponding to the early metabolic ebb phase, was independently associated with unfavorable functional outcome. Neither admission glucose nor glycemic variability were associated with outcome, and the conventional cutoff of > 180 mg/dL failed to differentiate risk groups. These observations suggest that timing and duration of early hyperglycemia provide clinically relevant insights beyond single or average glucose values. Nevertheless, it should be considered exploratory and hypothesis-generating, requiring prospective validation.

Stress-induced hyperglycemia has long been recognized as a part of the metabolic response to trauma; however, most studies have focused on admission glucose levels or daily averages. In particular, the cutoffs used to define stress hyperglycemia vary across different studies, underscoring the lack of a uniform predictive definition. In trauma studies high cutoffs (> 200 mg/dl) are frequently used [8, 9, 13, 35, 36]. Later studies showed that admission glucose more closely reflects injury severity rather than independent mortality risk [37], and that mean glucose may outperform admission value for mortality prediction [15]. For the association between glucose variability and mortality, supporting evidence remains limited, not least due to inconsistent sampling and short observation windows [38].

Data of this study demonstrated insufficiency of the previously described indices in predicting functional outcomes. While we observed higher average glucose levels in the unfavorable group, neither mean, TWAG nor admission values independently associated with outcomes in the multivariable analysis. In contrast, time-based indices, such TUHyperR, incorporate both the magnitude and duration of hyperglycemia, providing a more nuanced view of metabolic stress. Furthermore, by summarizing values within fixed 6-hour periods, it mitigates bias from irregular sampling and heterogeneous measurement timing and more accurately reflects sustained metabolic imbalance.

Temporal trend analysis confirmed a biphasic glucose pattern consistent with the classical ebb and flow phases of trauma metabolism [6, 10]. Consistent with previous trauma studies, glucose levels peaked after ICU admission and declined during the first 48 h, followed by stabilization thereafter. Prior studies showed continuously elevated levels in non-survivors [14, 19, 37]. In the present study, patients with unfavorable outcomes consistently maintained higher glucose concentrations during both phases. This suggests that incomplete metabolic normalization, rather than an exaggerated initial response, may underlie adverse outcomes. Mechanistically, early hyperglycemia reflects catecholamine- and cortisol-driven gluconeogenesis and insulin resistance [10, 12, 39]. Over time, as the flow phase begins, insulin secretion typically increases and gluconeogenesis decreases [12]. Persistent hyperglycemia thus may indicate ongoing excessive inflammation, endocrine dysregulation or an impaired ability to restore metabolic homeostasis.

Importantly, our study showed that sustained moderate hyperglycemia (> 140/160 mg/dL) during the ebb phase was stronger associated with poor functional outcome than short-term or extreme hyperglycemia (> 180 mg/dL). This finding aligns with evidence from other critically ill populations, showing that glycemic burden over time correlates with mortality and complications [7, 21, 24, 26, 27, 40]. Although the pathophysiological mechanisms are not yet fully understood, there are plausible links. Research, partly in vitro, suggests that persistent hyperglycemia might result in sustained oxidative stress, negatively affecting neutrophil activity, phagocytic capacity and impairing proliferation of fibroblasts [41]. Compromised tissue repair, immune dysfunction, increased complications and decreased functional outcome could be the consequences. Hence, focusing exclusively on single values or extreme cutoffs may overlook clinically relevant metabolic risks in trauma patients.

The current cutoff of 180 mg/dL for insulin therapy initiation in intensive care, as recommended by international guidelines [25], did not independently stratify outcome risk in our cohort. Our results indicate that outcome-relevant glycemic exposure begins at lower levels, particularly during the first 48 h. Given the small effect sizes observed for TUHyperR, these findings alone are unlikely to justify changes in the current treatment cutoffs. Instead, they suggest that early cumulative glycemic burden could provide prognostic information beyond ISS and conventional glucometrics and might therefore serve as an additional early risk marker of increased metabolic stress, a quality indicator of glycemic regulation and a complementary parameter to established risk scores for identifying high-risk patients.

Given the conflicting evidence on tight glycemic control in trauma [42–45] and the small effect sizes observed for TUHyperR in this study, routine lowering of the glucose treatment target is not supported by our data. Instead, vigilance for early, persistent moderate hyperglycemia, careful nutritional management, and context-specific interventions may be more appropriate. Nevertheless, future intervention studies should consider the investigation of early tighter blood glucose regulation on functional outcomes after polytrauma, possibly considering time in range using TUHyperR as a quality indicator. Furthermore, integration of time-based glycemic indices into expanding available electronic patient data management systems could facilitate real-time monitoring, thereby improve risk stratification and help to optimize individual metabolic management in ICU polytrauma care.

### Strengths and limitations

The strengths of this explorative study include the use of a time-unified approach to quantify hyperglycemia across distinct metabolic phases regardless of the varying measurement frequency and direct comparison with standard glycemic indices.

Nevertheless, this study has several limitations. Its retrospective, single-center and explorative design precludes causal inference and limits generalizability. The application of the ’Berlin definition’, exclusion of patients with diabetes, and the requirement of an ICU stay ≥ 72 h may have introduced selection and survivor bias by excluding patients discharged or dying early, thereby limiting the generalizability of our findings to broader trauma populations. However, these criteria were applied to ensure maximum comparability within the cohort and sufficient temporal resolution for glycemic analysis for both metabolic phases. Nonetheless, the resulting small sample size may have limited statistical power. Dietary and drug-related impacts including insulin, glucocorticoids, vasopressors, and nutrition were not systematically controlled due to a lack of consistent data. Although multivariable models and sensitivity analyses adjusted for various potential influencing factors, residual confounding by traumatic brain injury characteristics and other unmeasured factors cannot be excluded. TUHyperR was developed to mitigate direct effects of variable sampling by using fixed 6-hour periods. Nevertheless, it cannot be ruled out that the higher measurement frequency in the unfavorable GOS group might have resulted in a better representation of actual glucose trend, thereby influencing TUHyperR indirectly. As the effect sizes observed for TUHyperR were modest, the results should be interpreted as an additional risk signal rather than a strong stand-alone prognostic marker. Furthermore, despite adjusting for head injury severity, the observed associations do not imply causal independence from neurotrauma. Finally, the chosen hyperglycemic cutoffs were based on current guidelines and previous ICU literature; the study was not designed to identify an optimal glycemic target or establish therapeutic cutoffs. Therefore, the findings of this study should be considered hypothesis-generating and require validation in prospective multicenter studies.

## Conclusion

In this retrospective study, sustained moderate hyperglycemia within the first 48 h, quantified by a time-unified hyperglycemic rate, was independently associated with poor functional outcome after polytrauma. This underscores cumulative glucose exposure as a potentially relevant risk marker in the posttraumatic metabolic response and questions current glycemic risk cutoffs. As these findings are exploratory and hypothesis-generating, prospective studies are needed to clarify whether temporal glycemic indices, such as time-unified hyperglycemic rate, might support early risk stratification and optimize metabolic management in critically ill polytraumatized patients.

## Supplementary information

Below is the link to the electronic supplementary material.

Supplementary File 1 (PDF 250 KB)

## Data Availability

The datasets used and/or analyzed during the current study are available from the corresponding author on reasonable request.

## Abbreviations

ABG: Admission blood glucose
AIS: Abbreviated injury scale
aPTT: Activated partial thromboplastin time
ARDS: Acute respiratory distress syndrome
BGA: Blood gas analysis
BMI: Body mass index
CI: Confidence interval
COPD: Chronic obstructive pulmonary disease
CVG: Coefficient of variation for glucose
GCS: Glasgow coma scale
GOS: Glasgow outcome scale
ICU: Intensive care unit
INR: International normalized ratio
IQR: Interquartile range
ISS: Injury severity score
LOS: Length of stay
MBG: Mean blood glucose
OR: Odds ratio
SAPS II: Simplified acute physiology score II
SCCM: Society of critical care medicine
TUHyperR: Time-unified hyperglycemic rate
TWAG: Time-weighted average glucose
