# Supplementary material for: Association between sustained moderate hyperglycemia within first 48 hours and poor functional outcome after polytrauma: A retrospective cohort study
Source: Eur J Trauma Emerg Surg. 2026 Feb 23;52(1):63. doi: 10.1007/s00068-026-03099-3 (PMC12929354; doi:10.1007/s00068-026-03099-3)
Supplement: Supplementary file 1 — (PDF 250 KB) [file 68_2026_3099_MOESM1_ESM.pdf]

European Journal of Trauma and Emergency Surgery

Supplementary material

Association between sustained moderate hyperglycemia within first 48 hours and poor functional outcome after polytrauma: a retrospective cohort study

Matthias Manfred Deininger[1,*]; Paul Wassersteiner[1]; Nico Haehn[1]; Judith Huth[1]; Gernot Marx[1]; Christian David Weber[2]; Frank Hildebrand[2]; Tim-Philipp Simon[1]; Carina Benstoem[1], Thomas Breuer[1]

Affiliations:

[1] Department of Intensive Care Medicine, Faculty of Medicine, RWTH Aachen University, Aachen, Germany

[2] Department of Orthopaedic, Trauma and Reconstructive Surgery, Faculty of Medicine, RWTH Aachen University, Aachen, Germany

*Corresponding author: mdeininger@ukaachen.de

Table S1: Median AIS for the six ISS regions stratified by GOS outcome group

ISS region	Favorable (GOS >3)	Unfavorable (GOS ≤3)	p-value
Head and neck	3.0 (0.0-4.0)	4.0 (3.0-4.5)	0.125
Face	1.0 (0.0-2.0)	0.0 (0.0-2.5)	0.882
Chest	3.0 (2.0-3.0)	3.0 (3.0-4.0)	**0.020**
Abdomen	0.0 (0.0-3.0)	0.0 (0.0-2.0)	0.633
Extremities (including pelvis)	2.0 (2.0-3.0)	2.0 (2.0-3.0)	0.888
Soft tissue	0.0 (0.0-0.0)	0.0 (0.0-0.5)	0.589

Groupwise presentation of median (IQR) abbreviated injury scale (AIS) level for every of the six regions included in Injury severity score (ISS). Groups are split according to favorable (GOS >3) or unfavorable (GOS ≤3) outcome on hospital discharge. Significant p-values are shown in bold.

GOS: Glasgow outcome scale, IQR: Interquartile range

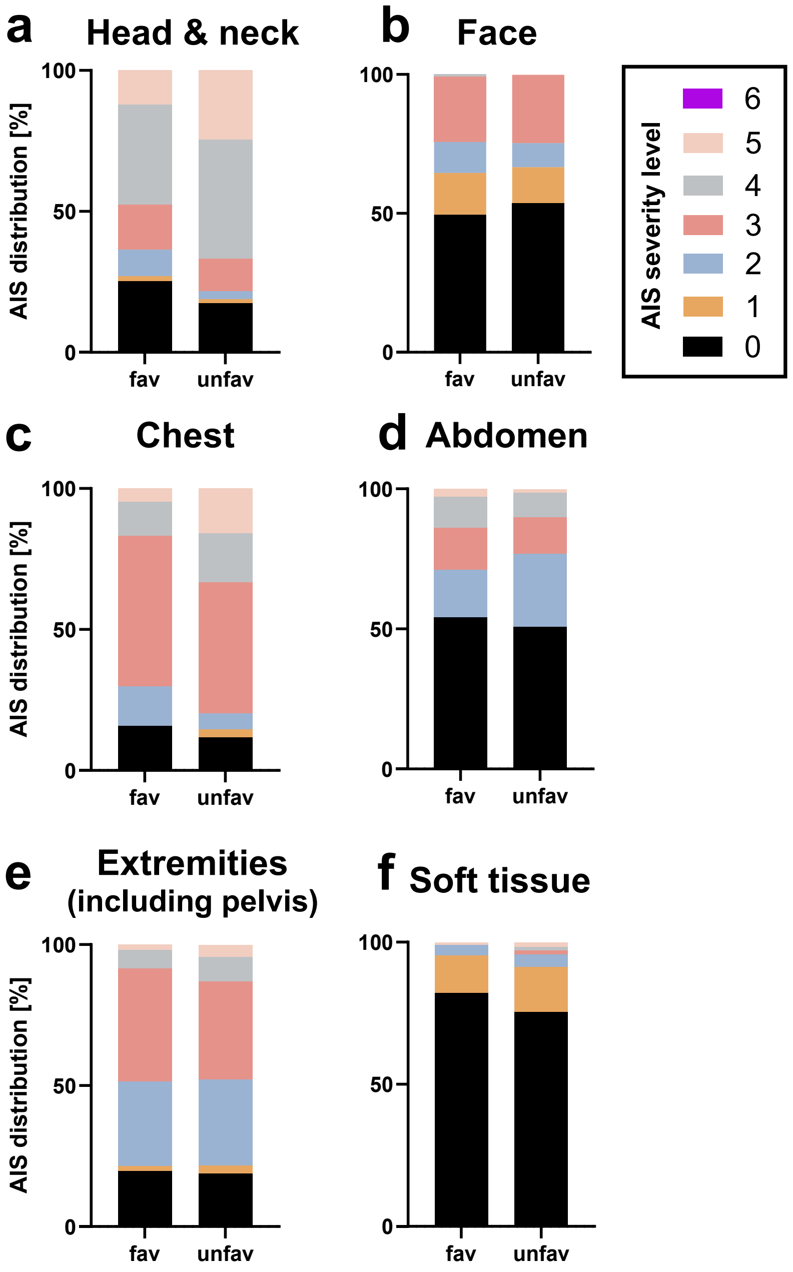

Fig. S1 Distribution of AIS severity levels for the six ISS regions, separated by GOS groups

The percentage distribution of AIS severity levels for each of the six ISS regions is shown separately for the two Glasgow Outcome Scale (GOS) groups (fav, favorable, GOS >3; unfav, unfavorable, GOS ≤3).

AIS: Abbreviated injury scale, ISS: Injury severity score

Table S2: Glycemic indices during ebb and flow phases stratified by outcome group – MBG data

Variable	Phases		p-value
	Ebb	Flow	
**Favorable (GOS >3)**			
MBG [mg/dl]	128.9 (119.7-143.0)	122.6 (114.1-131.9)	**<0.001**
**Unfavorable (GOS ≤3)**			
MBG [mg/dl]	137.2 (123.8-154.3)	127.1 (118.3-136.8)	**<0.001**

Data are analyzed separately for favorable and unfavorable outcome groups. For both groups, glycemic values for ebb and flow phases are examined. Data are shown as median (IQR). Significant p-values are shown in bold.

GOS: Glasgow outcome scale, IQR: Interquartile range, MBG: Mean blood glucose

Table S3: Univariable logistic regression for MBG prediction of unfavorable outcome

Variable	Univariate regression	
	**OR [95%-CI]**	**p-value**
MBG	1.015 [0.997-1.034]	0.106
MBG – ebb phase	1.017 [1.002-1.033]	**0.028**
MBG – flow phase	1.014 [0.996-1.033]	0.134

Glycemic indices that showed significant effects in the univariable analysis were included in separate multivariate models. Odds ratios (OR) and 95% confidence intervals (CI) are displayed. Significant p-values are shown in bold.

MBG: Mean blood glucose

Table S4: Glycemic indices stratified by metabolic phases

Variable	Hospital discharge Glasgow outcome scale		p-value
	**Favorable (>3)**	**Unfavorable (≤3)**	
**Ebb phase (≤48h)**			
MBG [mg/dl]	128.9 (119.7-143.0)	137.2 (123.8-154.3)	**0.012**
TWAG [mg/dL]	127.7 (116.8-141.3)	137.2 (123.2-150.7)	**0.020**
CVG [%]	14.0 (10.0-18.0)	14.0 (11.0-19.0)	0.233
TUHyperR (>140mg/dL) [%]	33.3 (12.5-62.5)	50.0 (25.0-87.5)	**0.005**
TUHyperR (>160mg/dL) [%]	12.5 (0.0-28.6)	25.0 (0.0-50.0)	**0.003**
TUHyperR (>180mg/dL) [%]	0.0 (0.0-12.5)	12.5 (0.0-14.3)	**0.010**
**Flow phase (>48h)**			
MBG [mg/dl]	122.6 (114.1-131.9	127.1 (118.3-136.8)	**0.039**
TWAG [mg/dL]	119.3 (111.4-128.1)	124.8 (117.1-135.0)	**0.008**
CVG [%]	15.0 (12.0-18.0)	16.0 (12.0-20.0)	0.355
TUHyperR (>140mg/dL) [%]	20.0 (9.5-34.7)	29.7 (14.3-45.7)	**0.009**
TUHyperR (>160mg/dL) [%]	4.9 (0.0-10.8)	8.0 (1.1-19.0)	0.050
TUHyperR (>180mg/dL) [%]	0.0 (0.0-3.6)	1.8 (0.0-6.3)	0.025

Data are analyzed separately for ebb (gray background) and flow (white background) phase. For both phases, glycemic values for favorable and unfavorable functional outcome groups were compared. Data are shown as median (IQR). Significant p-values are shown in bold.

CVG: Coefficient of variation for glucose, GOS: Glasgow outcome scale, IQR: Interquartile range, MBG: Mean blood glucose, TUHyperR: Time-unified hyperglycemic rate, TWAG: Time-weighted average glucose

Table S5: Multivariable logistic regression for prediction of unfavorable outcome – full ICU stay

Glycemic index	TUHyperR(>140mg/dL)		TUHyperR(>160mg/dL)	
**Variable**	**OR [95%-CI]**	**p-value**	**OR [95%-CI]**	**p-value**
Age	1.000 [0.982-1.018]	0.994	1.004 [0.988-1.021]	0.621
Sex	0.896 [0.417-1.926]	0.779	0.843 [0.395-1.800]	0.659
ISS	1.072 [1.031-1.113]	**<0.001**	1.072 [1.032-1.113]	**<0.001**
Glycemic index	1.016 [1.000-1.032]	0.050	1.015 [0.995-1.035]	0.149

All multivariable logistic regression models were adjusted for the baseline covariates age, sex and ISS. Odds ratios (OR) and 95% confidence intervals (CI) are displayed. Significant p-values are shown in bold.

ISS: Injury severity score, TUHyperR: Time-unified hyperglycemic rate

Table S6: Multivariable logistic regression for prediction of unfavorable outcome – ebb phase

Glycemic index	MBG		TWAG		TUHyperR(>140mg/dL)		TUHyperR(>160mg/dL)		TUHyperR(>180mg/dL)	
**Variable**	**OR [95%-CI]**	**p-value**	**OR [95%-CI]**	**p-value**	**OR [95%-CI]**	**p-value**	**OR [95%-CI]**	**p-value**	**OR [95%-CI]**	**p-value**
Age	1.002 [0.985-1.019]	0.803	1.002 [0.985-1.019]	0.806	1.000 [0.983-1.018]	0.989	1.001 [0.984-1.018]	0.886	1.006 [0.989-1.022]	0.502
Sex	0.829 [0.389-1.765]	0.626	0.825 [0.387-1.756]	0.617	0.841 [0.392-1.804]	0.656	0.816 [0.382-1.746]	0.600	0.788 [0.371-1.673]	0.535
ISS	1.073 [1.033-1.114]	**<0.001**	1.074 [1.035-1.116]	**<0.001**	1.075 [1.035-1.117]	**<0.001**	1.069 [1.029-1.111]	**<0.001**	1.070 [1.030-1.111]	**<0.001**
Glycemic index	1.016 [0.999-1.033]	0.069	1.015 [0.999-1.032]	0.070	1.015 [1.004-1.025]	**0.008**	1.016 [1.003-1.030]	**0.017**	1.015 [0.995-1.035]	0.141

All multivariable logistic regression models were adjusted for the baseline covariates age, sex and ISS. Odds ratios (OR) and 95% confidence intervals (CI) are displayed. Significant p-values are shown in bold.

ISS: Injury severity score, MBG: Mean blood glucose, TUHyperR: Time-unified hyperglycemic rate, TWAG: Time-weighted average glucose

Table S7: Multivariable logistic regression for prediction of unfavorable outcome – flow phase

Glycemic index	TUHyperR(>140mg/dL)	
**Variable**	**OR [95%-CI]**	**p-value**
Age	1.001 [0.983-1.019]	0.936
Sex	0.899 [0.418-1.935]	0.786
ISS	1.071 [1.031-1.112]	**<0.001**
Glycemic index	1.014 [0.999-1.030]	0.065

The multivariable logistic regression model was adjusted for the baseline covariates age, sex and ISS. Odds ratios (OR) and 95% confidence intervals (CI) are displayed. Significant p-values are shown in bold.

ISS: Injury severity score, TUHyperR: Time-unified hyperglycemic rate

Table S8: Sensitivity analysis using multivariable logistic regression adjusting for head injury in ebb phase (binary)

	TUHyperR(>140mg/dL)				TUHyperR(>160mg/dL)			
**adjusting for head injury**	**Any head injury**		**Severe head injury**		**Any head injury**		**Severe head injury**	
**Variable**	**OR [95%-CI]**	**p-value**	**OR [95%-CI]**	**p-value**	**OR [95%-CI]**	**p-value**	**OR [95%-CI]**	**p-value**
Age	1.000 [0.983-1.017]	0.995	1.000 [0.982-1.017]	0.965	1.001 [0.984-1.018]	0.901	1.001 [0.984-1.018]	0.942
Sex	0.834 [0.389-1.790]	0.642	0.818 [0.380-1.760]	0.608	0.810 [0.379-1.734]	0.588	0.797 [0.371-1.708]	0.559
ISS	1.074 [1.034-1.117]	**<0.001**	1.072 [1.031-1.115]	**<0.001**	1.069 [1.028-1.110]	**<0.001**	1.066 [1.026-1.108]	**0.001**
Head injury (AIS>0)	1.220 [0.535-2.780]	0.636			1.279 [0.565-2.891]	0.555		
Severe head injury (AIS ≥ 3)			1.601 [0.760-3.373]	0.216			1.627 [0.775-3.415]	0.199
Glycemic index	1.014 [1.003-1.025]	**0.012**	1.014 [1.003-1.025]	**0.012**	1.016 [1.002-1.029]	**0.022**	1.016 [1.002-1.029]	**0.022**

All multivariable logistic regression models were adjusted for the baseline covariates age, sex and ISS as well as head injury (AIS>0) or severe head injury (AIS ≥ 3). Odds ratios (OR) and 95% confidence intervals (CI) are displayed. Significant p-values are shown in bold.

AIS: Abbreviated injury scale, ISS: Injury severity score, TUHyperR: Time-unified hyperglycemic rate

Table S9: Sensitivity analysis using multivariable logistic regression adjusting for head injury severity in ebb phase (continuous)

	TUHyperR(>140mg/dL)		TUHyperR(>160mg/dL)	
**Variable**	**OR [95%-CI]**	**p-value**	**OR [95%-CI]**	**p-value**
Age	1.000 [0.982-1.017]	0.957	1.000 [0.983-1.018]	0.959
Sex	0.842 [0.392-1.806]	0.658	0.820 [0.383-1.754]	0.609
ISS	1.071 [1.029-1.114]	**<0.001**	1.064 [1.023-1.107]	**0.002**
Head injury severity (AIS)	1.086 [0.896-1.318]	0.400	1.097 [0.906-1.329]	0.343
Glycemic index	1.014 [1.003-1.025]	**0.012**	1.016 [1.002-1.029]	**0.022**

All multivariable logistic regression models were adjusted for the baseline covariates age, sex and ISS as well as head injury severity represented by AIS level. Odds ratios (OR) and 95% confidence intervals (CI) are displayed. Significant p-values are shown in bold.

AIS: Abbreviated injury scale, ISS: Injury severity score, TUHyperR: Time-unified hyperglycemic rate

Table S10: Sensitivity analysis using multivariable logistic regression adjusting for chest severity in ebb phase (continuous)

	TUHyperR(>140mg/dL)		TUHyperR(>160mg/dL)	
**Variable**	**OR [95%-CI]**	**p-value**	**OR [95%-CI]**	**p-value**
Age	1.001 [0.984-1.018]	0.925	1.002 [0.985-1.019]	0.825
Sex	0.845 [0.393-1.818]	0.667	0.820 [0.383-1.756]	0.609
ISS	1.067 [1.024-1.112]	**0.002**	1.062 [1.019-1.107]	**0.004**
Chest injury severity (AIS)	1.131 [0.869-1.473]	0.360	1.108 [0.852-1.441]	0.443
Glycemic index	1.015 [1.004-1.026]	**0.007**	1.017 [1.003-1.030]	**0.015**

All multivariable logistic regression models were adjusted for the baseline covariates age, sex and ISS as well as chest injury severity represented by AIS level. Odds ratios (OR) and 95% confidence intervals (CI) are displayed. Significant p-values are shown in bold.

AIS: Abbreviated injury scale, ISS: Injury severity score, TUHyperR: Time-unified hyperglycemic rate
